# Scalp and Neck Metastasis From HER2‐Positive Gastric Cancer: A Case Report and Literature Review

**DOI:** 10.1155/crom/9995795

**Published:** 2026-05-23

**Authors:** Ricardo Fernández-Ferreira, Jesus Montaño-Hernández, Rafael-Garza Cantú-Pedraza, Fernanda Delgado-Gutierrez, Renato-Adrian Flores-Vazquez, Mauricio-Lohengrin Durán-Aceves, Verónica Bautista-Piña, Rosa-María Vicuña-González, María-del-Carmen Burguete-Bojalil, Giovana-Ivette De-Lucio-Chavero

**Affiliations:** ^1^ Oncology Medical (Oncology Department), Pemex (Mexico), Mexico City, Mexico; ^2^ Oncology Medical (Oncology Department), Medica Sur, Mexico City, Mexico; ^3^ Oncology Department, National Autonomous University of Mexico, Mexico City, Mexico, unam.mx; ^4^ Pathology Department, Pemex (Mexico), Mexico City, Mexico

**Keywords:** gastric cancer, HER2-positive, scalp, skin metastasis, trastuzumab

## Abstract

**Background:**

Cutaneous metastasis of gastric cancer is a rare manifestation (2.6%) of advanced gastrointestinal cancers and occurs in less than 1% of upper GI tract malignancies. The nodular type is the most common type. Tissue diagnosis and anatomic localization of the primary tumor are best obtained by upper gastrointestinal endoscopy. However, when the patient presents with dermatological and scalp lesions, the procedure is to take a targeted biopsy of said lesions.

**Case Summary:**

A 60‐year‐old female with advanced disease (Stage IV HER2‐positive gastric cancer) was diagnosed due to skin metastases: erythematous alopecic skin lesions on the central scalp, as well as a raised, erythematous, indurated, nontender, irregular 6 × 8 cm lesion on the cervicodorsal region. Treatment with capecitabine + oxaliplatin + trastuzumab every 3 weeks was initiated. By the sixth cycle, the scalp lesion had resolved completely, and the cervical lesion had reduced to 3 × 4 cm. After 1 year of treatment, the lesions continued to show a favorable response.

**Conclusions:**

To the best of our knowledge, this is the second reported case of cutaneous metastases from HER2‐positive gastric cancer. Fewer than 20 cases involving metastases to the scalp and neck region have been reported in the English‐language literature.

## 1. Introduction

Gastric cancer (GC) is the fifth leading cause of global cancer incidence and mortality in 2022. The incidence of GC is highest in Eastern Asia (Mongolia, China, South Korea, and Japan), the Andean regions of South America, and Eastern Europe [1]. Cutaneous metastatic (CM) lesions often occur in the final stage of cancer. CM from internal malignancy is relatively uncommon, with a reported frequency varying between 0.7% and 9% [2].

CM of GC is a rare manifestation (2.6%) of advanced gastrointestinal cancers and occurs in less than 1% of upper GI tract malignancies [3, 4]. The scalp is an unusual site of CM. Brownstein and Helwig previously reported that scalp metastasis accounts for 4% of all skin metastases [5].

GC that is metastatic to the scalp and neck is extremely rare, with only a few cases reported to date. It usually presents as single or multiple firm scalp nodules [6]. Neoplastic alopecia is a well‐recognized but rare presentation, manifesting as single or multiple areas of cicatricial alopecia, which typically appear suddenly and grow rapidly [7].

To the best of our knowledge, fewer than 75 cases of CMs from GC and fewer than 20 cases involving the scalp and neck region have been reported in the English‐language literature. Here, we report a rare case of scalp and CMs from gastric signet ring cell carcinoma (SRCC), human epidermal growth factor receptor 2 (HER2)‐positive, and review the relevant literature.

### 1.1. CARE Checklist Statement

The authors have read the CARE Checklist, and the manuscript was prepared and revised according to the CARE Checklist. Summarizing why this case is unique: References [2–7].

## 2. Case Report

The patient was a 60‐year‐old female from Mexico, with a family history of GC in her mother and a personal history of smoking and alcohol consumption.

**Figure 1:**
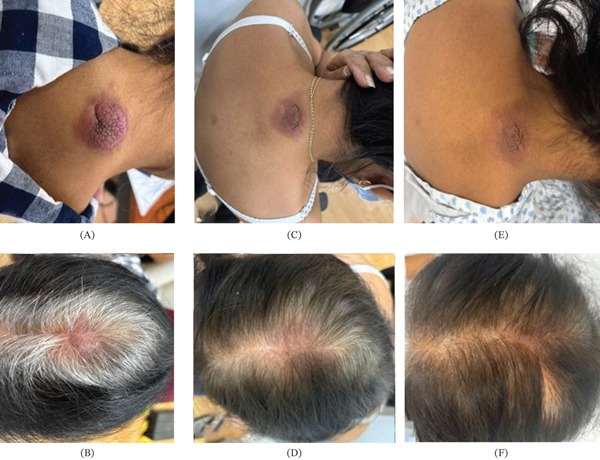
(A) Erythematous, sclerodermoid, nontender, irregular lesions measuring 6 × 8 cm located in the cervicodorsal region, (B) as well as erythematous, nodular, alopecic lesions on the central scalp. (C) By the sixth cycle of chemotherapy, the cervical lesion had decreased to 3 × 4 cm, and (D) the scalp lesion had completely resolved . (E) After 1 year of treatment, the neck lesion remains erythematous, flat, and measures 3 × 3 cm. (F) The scalp lesion has disappeared completely, with only residual erythema and hair regrowth observed at the site.

She went to an emergency consultation in 2024 due to presenting with left pleural effusion, requiring thoracentesis and supplemental oxygen. On physical examination, erythematous, indurated, nontender, irregular lesions measuring 6 × 8 cm were observed on the cervicodorsal region (Figure 1A), as well as erythematous alopecic skin lesions on the central scalp (Figure 1B).

**Figure 2:**
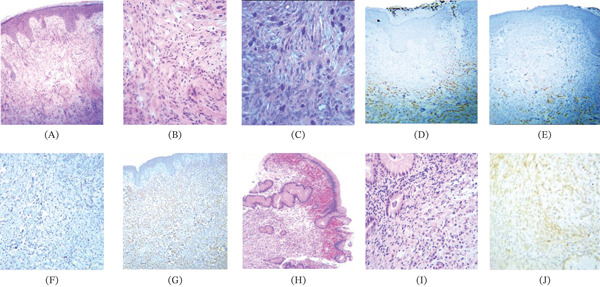
(A) Hematoxylin and eosin staining of the skin biopsy showing a diffuse epithelial neoplasm within a desmoplastic stroma infiltrating the papillary and reticular dermis. (B) The neoplastic cells contain intracytoplasmic vacuoles that displace the nucleus toward the periphery, giving the cells their characteristic “signet ring” appearance. (C) The vacuoles of the neoplastic cells are PAS‐positive. Immunohistochemistry shows (D) CK7 positivity in the cytoplasmic membranes of neoplastic cells, (E) CK20 positivity in the cytoplasmic membranes of neoplastic cells, (F) CDX2 weak and irregular nuclear positivity in neoplastic cells, and (G) CK19 positivity in the cytoplasmic membranes of neoplastic cells. (H) In the gastric ulcer biopsy, a diffuse infiltrating neoplasm within a desmoplastic stroma is identified in the submucosa. (I) Signet ring cells are observed in greater detail. (J) Immunohistochemistry demonstrates HER2 positivity in the neoplastic cells.

Following resolution of the pleural effusion, a skin biopsy was performed, revealing metastatic diffuse adenocarcinoma with signet ring cells. Subsequent endoscopy identified a gastric fundus ulcer, and biopsy confirmed a poorly differentiated diffuse adenocarcinoma with signet ring cells and HER2‐positive (Figure 2).

Her tomography of the chest, abdomen, and pelvis with contrast reported metastasis to the lungs, multiple subcutaneous lesions at the scalp level, and a dermal lesion in the cervicodorsal region.

Advanced disease (Stage IV GC) was diagnosed due to skin metastases, and palliative treatment with capecitabine 1000 mg/m^2^ + oxaliplatin 130 mg/m^2^ + trastuzumab 6 mg/kg every 3 weeks was initiated.

After the first two cycles, significant regression of the skin lesions and pleural effusion was observed, with the patient no longer requiring supplemental oxygen. By the sixth cycle, the scalp lesion had resolved completely (Figure 2D), and the cervical lesion had reduced to 3×4 cm (Figure 2C). After 1 year of treatment, the lesions continued to show a favorable response (Figure 1E,F). Therapy was eventually discontinued due to massive and fulminant pulmonary thromboembolism, as well as pleural effusion. He died a month later from acute respiratory failure.

## 3. Discussion

GC most commonly metastasizes to the lymph nodes (50%), liver (48%), peritoneum (32%), lung (15%), and bone (11.3%). Metastases occur less frequently in the ovaries (5.4%–9.7%), central nervous system (< 1%), breast (0.5%–1.3%), cutaneous sites of the head and neck (5%–9%), and the scalp (2%) [8, 9].

Scalp metastases are rare, occurring in fewer than 2% of patients with metastatic malignancies. Lung cancer (23.53%) is the most common primary tumor metastasizing to the scalp, followed by colorectal cancer (11.76%), hepatocellular carcinoma (7.84%), and breast cancer (7.84%). Notably, metastases of unknown primary account for 29.41% of all scalp metastases [10, 11].

We conducted a review of the English‐language literature, identifying 18 case reports of GC metastatic to the scalp or neck (Table 1). The mean age was 55 years (range 33–73), and 50% of cases occurred in women. Neck involvement was reported in five cases (28%), while scalp involvement was observed in 13 cases (72%) [3, 4, 6, 7, 12–24].

**Table 1:** Scalp and neck metastasis from gastric cancer.

Source	Age	Sex	Site(s) of cutaneous metastasis	Type	Histology/immunohistochemistry	Time of occurrence (months)	Management	Prognostic
Chen et al. (2021)[11]	69	M	Forehead, back, neck, and arms	Nodular	Adenocarcinoma with signet ring cells	1	Chemotherapy	Unknown/> 3 months
Yang et al. (2020)[12]	57	M	Neck and shoulder	Sclerodermoid	Adenocarcinoma/HER2−	1	Chemotherapy	Unknown
Yao et al. (2023)[13]	61	M	Left groin and scalp	Nodular sclerodermoid	Adenocarcinoma (HER2−)	1	Immunotherapy//chemotherapy (> 3 months)	Unknown
Gündüz et al. (2017)[14]	57	F	Face, neck, and shoulders	Nodular	Adenocarcinoma with signed ring cells	0	Chemotherapy and surgery	Unknown
Ahmad et al. (2015)[15]	49	F	Scalp, face, upper limbs, shoulder, back, and chest	Nodular	Adenocarcinoma	1	Chemotherapy	> 3 months
Arslan et al. (2014)[16]	52	M	Face and scalp	Nodular	Adenocarcinoma with signed ring cells//HER2+	1	Chemotherapy/immunotherapy	6 months
Früh et al. (2005)[17]	60	M	Scalp	Nodular	Adenocarcinoma	1	Resection	> 5 years
Sakaki et al. (1979)[18]	53	F	Scalp	Nodular	Adenocarcinoma	1	N/A	5 days
Kim et al. (1999)[7]	36	F	Scalp	Scleroderma	Adenocarcinoma with signed ring cells	2	Chemotherapy	Unknown (3 months)
Lifshitz et al. (2005)[4]	73	M	Upper forehead and scalp	Nodular	Adenocarcinoma	4	Chemotherapy	> 12 months
Frey et al. (2009)[3]	54	M	Scalp	Nodular	Adenocarcinoma	1	Chemotherapy	4 months
Du et al. (2015)[6]	41	F	Scalp	Sclerodermoid	Adenocarcinoma with signed ring cells/HER2−	5	Chemotherapy/radiotherapy	6 months
Kim et al. (2014)[19]	33	F	Scalp	Nodular	Adenocarcinoma with signed ring cells	3	Chemotherapy	Unknown
Kohno et al. (1983)[20]	37	F	Scalp	Sclerodermoid	Adenocarcinoma	1	Surgery and chemotherapy	Unknown
Karakoca et al. (2010)[21]	68	F	Neck	Nodular	Adenocarcinoma	1	Unknown	Unknown
Hayashi et al. (2009)[22]	61	F	Neck	Nodular	Adenocarcinoma	1	Unknown	Unknown
Ryu et al. (2021)[23]	60	M	Scalp	Nodular	Adenocarcinoma	1	Surgery	12 months
Menghani et al. (2020)[24]	69	M	Scalp	Sclerodermoid	Adenocarcinoma	1	Chemotherapy	Unknown

### 3.1. Clinical Characteristics of CMs From GC

In advanced disease, common signs and symptoms include dysphagia, asthenia, indigestion, vomiting, weight loss, early satiety, and/or iron deficiency anemia [25, 26].

The diagnosis of GC may be suspected in patients with abdominal pain or weight loss and a history of gastric ulcer or because of findings on upper endoscopy or radiographic imaging (e.g., abdominal computed tomography [CT] or barium studies) [27].

Scalp metastatic lesions may grow unnoticed for a long period of time, manifesting as atypical nodules or plaques or alopecia neoplastica in even rarer cases. As scalp metastasis lacks characteristic clinical presentations, it is often overlooked as an ordinary skin disease, which is the main reason for delayed diagnosis and treatment [7].

They are most commonly nodular (46%), hyperemic or hyperpigmented, asymptomatic lesions associated with hair follicle loss [28]; the second most common clinical presentation in the 28% of cases is an erysipelas‐like lesion (an erythematous, hyperemic area, painful to superficial palpation, which does not respond to antibiotic therapy in patients with a history of cancer) [29–31]. It can be confused with neurofibroma, ulcers (10%), and mixed [32]. Sister Maria Jose′s nodule represents intra‐abdominal CMs but is not specific for GC [32].

From the review we did (see Table 1), the form of presentation was nodular in 12 (67%) of the cases, sclerodermoid in five (27%), and mixed (sclerodermoid and nodular) in one (6%), with a time of occurrence of metastatic disease and skin in approximately 1.5 months [3, 4, 6, 7, 12–24].

The case we present is that of a 60‐year‐old woman with nodular GC metastatic to the scalp and sclerodermoid to the neck, with the occurrence of the metastatic event at the time of diagnosis.

### 3.2. Diagnosis

Tissue diagnosis and anatomic localization of the primary tumor are best obtained by upper gastrointestinal endoscopy. However, when the patient presents with dermatological and scalp lesions, the procedure is to take a targeted biopsy of said lesions [33].

### 3.3. Histopathology

Gastric adenocarcinomas have historically been divided into two distinct histomorphologic subtypes (Lauren, 1965): intestinal in 44%–77% (i.e., gland‐forming) and diffuse in 9%–38% (composed of discohesive cells) and mixed in 14%–17%, which have a distinct morphologic appearance, epidemiology, pathogenesis, and genetic profile. The most recent World Health Organization (WHO, 2019) classification of tumors of the digestive tract recognizes several important histologic types of malignant epithelial tumors, which include gland‐forming types (tubular, papillary mucinous, and mixed) and poorly cohesive types (including the signet ring phenotype). HER2 expression according to tumor subtype (Lauren classification) has been reported as 16.8% in the intestinal subtype, 2.3% in the diffuse subtype, and 8.4% in the mixed subtype [10, 33–34].

GC often displays heterogeneity of the *HER2* genotype and phenotype, which might be partly accountable for testing inaccuracy. Approximately 10%–20% of gastric adenocarcinomas have *HER2* gene amplification, which results in overexpression of HER2. HER2 positivity varied by tumor site, with higher rates of HER2 positivity in gastroesophageal junction (GEJ) adenocarcinoma than in stomach cancer in this study (14.6% vs. 7.0%, respectively; *p* < 0.01) [35].

SRCC is a histologic subtype of GCs that accounts for 8%–30% of all stomach cancers and is usually associated with advanced‐stage cancer and recognized as a prognostic factor [27]. SRCC has a similar metastatic spread pattern to the other GCs and tends to metastasize particularly to the peritoneum and intra‐abdominal organs (liver, ovaries, etc.). CMs of SRCC are also very rare and may present as nodular lesions, such as in this case, mimicking benign or non‐neoplastic or benign entities, such as erysipelas, scars, or contact dermatitis, complicating the diagnostic process in the absence of a known primary tumor. According to our review (see Table 1), signet ring cell adenocarcinoma was reported in six (30%) cases, as well as HER2‐negative in three cases and one case with HER2‐positive, like the case we present [3, 4, 6, 7, 12–24].

### 3.4. Imagen

For patients with a suspected GC, contrast‐enhanced (CT) imaging provides information about the primary tumor, and it can also visualize low‐volume ascites, peritoneal metastases, liver, lung, ovarian, skin, scalp metastases, and perigastric and distant nodal disease [33]. Magnetic resonance imaging (MRI) is an auspicious method for depicting various gastric wall layers and the differentiation of tumor tissue from fibrosis [36].

The role of 18‐fluorodeoxyglucose (FDG)‐PET in the staging evaluation of GC continues to evolve. Previously, we used it liberally in any patient with ≥ T2N0 disease despite a negative CT. With the current high quality of contrast‐enhanced CT imaging, we have found a decreasing yield with the expanded use of PET [37]. This is particularly true for diffuse‐type tumors, where significant numbers of patients have tumors that are not FDG‐avid. Further, for patients with signet ring cell histology, the peritoneum is the most common site of metastatic disease, a site that we find better assessed by laparoscopy with washings. Generally, we reserve PET‐CT for those patients who have equivocal findings on CT imaging or patients with clinical indications of possible metastatic disease and otherwise negative imaging (e.g., radiographically occult metastatic lesions in approximately 10% of patients with locally advanced GC (≥ T3 or ≥ N1 disease) [37, 38].

### 3.5. Treatment

In the open‐label Phase III ToGA trial, the addition of trastuzumab to cisplatin‐based chemotherapy plus a fluoropyrimidine (capecitabine or infusional 5‐FU) improved overall survival (OS) and progression‐free survival (PFS) and was well tolerated. At a median follow‐up of 17–19 months, trastuzumab in combination with chemotherapy significantly improved OS (median 14 vs. 11 months; HR 0.74, 95% CI 0.60–0.91), PFS (median 6.7 vs. 5.5 months; HR 0.71, 95% CI 0.59–0.86), and objective response rate (47% vs. 35%) [35].

In an exploratory subgroup analysis, trastuzumab improved OS among patients with HER2 IHC 3+ tumors (HR 0.66, 95% CI 0.50–0.87) [35]. Current evidence supports the addition of trastuzumab to fluoropyrimidine and oxaliplatin‐based chemotherapy rather than to fluoropyrimidine and cisplatin‐based chemotherapy, as this approach is associated with improved OS and a more favorable toxicity profile [39].

In a meta‐analysis of prospective and retrospective studies including 557 patients with advanced HER2‐positive gastric and GEJ adenocarcinoma, trastuzumab was evaluated in combination with various chemotherapy regimens as first‐line therapy. Compared with the ToGA regimen (trastuzumab, cisplatin, and a fluoropyrimidine), the combination of trastuzumab plus oxaliplatin and either capecitabine or 5‐FU was associated with improved OS (median 20.7 vs. 16 months; HR 0.75, 95% CI 0.59–0.99) and lower toxicity [40].

### 3.6. Prognosis

CM in GC is generally associated with a poor prognosis. The interval between the diagnosis of GC and the appearance of CMs ranges from 1 to 3 years, with a maximum reported interval of up to 15 years [12, 41].

The average OS after the diagnosis of CM is approximately 6 months. In more than 50% of cases, patients present with additional metastatic sites at the time of diagnosis [31, 41–44].

We report a case of HER2‐positive gastric adenocarcinoma with signet ring cell features, with metastases to the neck and scalp presenting as nodular and sclerodermoid lesions, which showed a favorable response to chemotherapy combined with anti‐HER2 therapy, achieving an OS of 1 year despite the poor histological and clinical prognosis.

## 4. Conclusions

GC with metastases to the scalp and neck is extremely rare, with only a limited number of cases reported to date. To the best of our knowledge, fewer than 75 cases of CMs from GC involving the head and neck region have been reported in the English literature. Anti‐HER2 therapy with trastuzumab has demonstrated clinical benefit in patients with CMs, particularly those involving the scalp.

### 4.1. CARE Checklist Statement

The authors have read the CARE Checklist, and the manuscript was prepared and revised according to the CARE Checklist. Discussion of the relevant medical literature, References 12–24.

## Author Contributions

All authors contributed to the conception of the case, analysis, and critical revision of the content. Ricardo Fernández‐Ferreira, Rafael‐Garza Cantú‐Pedraza, Verónica Bautista‐Piña, and Rosa‐María Vicuña‐González provided clinical care for the patient. Ricardo Fernández‐Ferreira, Jesus Montaño‐Hernández, María‐del‐Carmen Burguete‐Bojalil, and Giovana‐Ivette De‐Lucio‐Chavero wrote the manuscript. Ricardo Fernández‐Ferreira, Fernanda Delgado‐Gutierrez, Renato‐Adrian Flores‐Vazquez, and Mauricio‐Lohengrin Durán‐Aceves were the attending consultants. Jesus Montaño‐Hernández reviewed the final draft of the manuscript. Jesus Montaño‐Hernández, Verónica Bautista‐Piña, and Rosa‐María Vicuña‐González carried out an exhaustive review of the histopathological characteristics of cancer and an analysis of the article.

## Funding

We did not receive any funding for the preparation of this case report.

## Disclosure

All authors gave their final approval of the version to be published. All authors agree to be responsible for all aspects of the job to ensure that questions related to the accuracy or completeness of any part of it are properly investigated and resolved. The authors have read the CARE Checklist, and the manuscript was prepared and revised according to the CARE Checklist.

## Ethics Statement

This retrospective review of patient data did not require ethical approval in accordance with local/national guidelines.

## Consent

All the patients allowed personal data processing. Written informed consent was obtained from the patient for the publication of the details of their medical case and any accompanying images.

## Conflicts of Interest

The authors declare no conflicts of interest.

## Supporting information


**Supporting Information** Additional supporting information can be found online in the Supporting Information section. **Supporting Information.**

## Data Availability

The data that support the findings of this study are available upon request from the corresponding author. The data are not publicly available due to privacy or ethical restrictions.

## References

[1] Siegel R. L. , Giaquinto A. N. , and Jemal A. , Cancer Statistics, 2024, Ca: A Cancer Journal For Clinicians. (2024) 74, no. 1, 12–49, 10.3322/caac.21820, 38230766. 38230766

[2] Hu S. C. , Chen G. S. , Wu C. S. , Chai C. Y. , and Chen W. T. , Rates of Cutaneous Metastases From Different Internal Malignancies: Experience From a Taiwanese Medical Center, Journal of the American Academy of Dermatology. (2009) 60, no. 3, 379–387, 10.1016/j.jaad.2008.10.007, 2-s2.0-60149092889. 19056145

[3] Frey L. , Vetter-Kauczok C. , Gesierich A. , Bröcker E. B. , and Ugurel S. , Cutaneous Metastases as the First Clinical Sign of Metastatic Gastric Carcinoma, JDDG: Journal der Deutschen Dermatologischen Gesellschaft. (2009) 7, no. 10, 893–895, 10.1111/j.1610-0387.2009.07102.x, 2-s2.0-70349922907, 19538484. 19538484

[4] Lifshitz O. H. , Berlin J. M. , Taylor J. S. , and Bergfeld W. F. , Metastatic Gastric Adenocarcinoma Presenting as an Enlarging Plaque on the Scalp, Cutis. (2005) 76, no. 3, 194–196, 16268264. 16268264

[5] Brownstein M. H. and Helwig E. B. , Patterns of Cutaneous Metastasis, Archives of Dermatology. (1972) 105, no. 6, 862–868, 10.1001/archderm.1972.01620090034008, 2-s2.0-0015350235. 5030236

[6] Du C. , Hong R. , Liu Y. , Wang J. , and Zhang H. , Scalp Metastasis From Gastric Cancer: A Case Report and Literature Review, Oncology Letters. (2015) 9, no. 2, 641–644, 10.3892/ol.2014.2708, 2-s2.0-84918836279. 25624893 PMC4301561

[7] Kim H. J. , Min H. G. , and Lee E. S. , Alopecia Neoplastica in a Patient With Gastric Carcinoma, British Journal of Dermatology. (1999) 141, no. 6, 1122–1124, 10.1046/j.1365-2133.1999.03217.x, 2-s2.0-0033392310. 10606865

[8] Allum W. , Lordick F. , Alsina M. , Andritsch E. , and Ba-Ssalamah A. , ECCO Essential Requirements for Quality Cancer Care: Oesophageal and Gastric Cancer, Critical Reviews in Oncology/Hematology. (2018) 122, 179–193, 10.1016/j.critrevonc.2017.12.019, 2-s2.0-85044134065. 29458786

[9] Wen L. , Li Y. Z. , Zhang J. , Zhou C. , Yang H. N. , Chen X. Z. , Xu L. W. , Kong S. N. , Wang X. W. , and Zhang H. M. , Clinical Analysis of Bone Metastasis of Gastric Cancer: Incidence, Clinicopathological Features and Survival, Future Oncology. (2019) 15, no. 19, 2241–2249, 10.2217/fon-2019-0039, 2-s2.0-85069482563, 31215231. 31215231

[10] Chiu C. S. , Lin C. Y. , Kuo T. T. , Kuan Y. Z. , and Chen M. J. , Malignant Cutaneous Tumors of the Scalp: A Study of Demographic Characteristics and Histologic Distributions of 398 Taiwanese Patients, Journal of the American Academy of Dermatology. (2007) 56, no. 3, 448–452, 10.1016/j.jaad.2006.08.060, 2-s2.0-33847034786. 17141358

[11] Chen J. W. , Zheng L. Z. , Xu D. H. , and Lin W. , Extensive Cutaneous Metastasis of Recurrent Gastric Cancer: A Case Report, World Journal of Clinical Cases. (2021) 9, no. 22, 6575–6581, 10.12998/wjcc.v9.i22.6575. 34435028 PMC8362582

[12] Yang S. , Liu X. L. , Guo X. L. , Song B. , and Li S. Z. , Solitary Metastasis to the Skin and Colon From Gastric Cancer After Curative Gastrectomy and Chemotherapy: A Case Report, Medicine. (2020) 99, no. 31, e21532, 10.1097/MD.0000000000021532. 32756202 PMC7402901

[13] Yao S. , Zhou P. , Li Y. , and Li Q. , Case Report: A Case of Delayed Cutaneous Metastases From Signet-Ring Cell Mixed-Type Gastric Cancer, Frontiers In Oncology. (2023) 13, no. 13, 1105080, 10.3389/fonc.2023.1105080, 36923441. 36923441 PMC10010164

[14] Gündüz Ö. , Emeksiz M. C. , Atasoy P. , Kidir M. , and Yalçin S. , Signet-Ring Cells in the Skin: A Case of Late-Onset Cutaneous Metastasis of Gastric Carcinoma and a Brief Review of Histological Approach, Dermatology Reports. (2017) 8, no. 1, 10.4081/dr.2016.6819. PMC5225968 28326183

[15] Ahmad B. , Pierson N. , Adnan M. M. , Phan M. , and Jenkins J. , Distant Skin Metastases as Primary Presentation of Gastric Cancer, Journal of Community and Supportive Oncology. (2015) 13, no. 4, 156–158, 10.12788/jcso.0127. 26102608

[16] Arslan D. , Uysal M. , Tatlı A. M. , Gunduz S. , and Goksu S. S. , Her-2 Positive Gastric Cancer Presented With Thrombocytopenia and Skin Involvement: A Case Report, Case Reports in Oncological Medicine. (2014) 2014, 194636, 10.1155/2014/194636. 25045559 PMC4090488

[17] Früh M. , Ruhstaller T. , Neuweiler J. , and Cerny T. , Resection of Skin Metastases From Gastric Carcinoma With Long-Term Follow-Up: An Unusual Clinical Presentation, Onkologie. (2005) 28, no. 1, 38–40, 10.1159/000082266, 2-s2.0-12544253576. 15604627

[18] Sakaki S. , Mori Y. , Matsuoka K. , Ohnishi T. , and Bitoh S. , Metastatic Dural Carcinomatosis Secondary to Gastric Cancer, Neurologia Medico-Chirurgica. (1979) 19, no. 1, 39–44, 10.2176/nmc.19.39, 2-s2.0-0018404659. 84354

[19] Kim J. H. , Kim M. J. , Sim W. Y. , and Lew B. L. , Alopecia Neoplastica due to Gastric Adenocarcinoma Metastasis to the Scalp, Presenting as Alopecia: A Case Report and Literature Review, Annals of Dermatology. (2014) 26, no. 5, 624–627, 10.5021/ad.2014.26.5.624, 2-s2.0-84907630117. 25324657 PMC4198592

[20] Kohno A. , Saruta T. , and Kimura H. , A Case of Alopecia Neoplastica due to Cutaneous Metastasis From Stomach, Rhinsho Derm. (1983) 25, 334–335.

[21] Karakoca Y. , Aslan C. , Erdemir A. T. , Kiremitci U. , and Gurel M. S. , Neurofibroma Like Nodules on Shoulder: First Sign of Gastric Adenocarcinoma, Dermatology Online Journal. (2010) 16, no. 5. 20492829

[22] Hayashi K. , Yamamoto T. , Oyama K. , Nagai T. , and Tsuboi R. , Epidermotropic Skin Metastasis From Gastric Cancer: Immunohistochemical Analysis Using Cytokeratins, Clinical and Experimental Dermatology. (2009) 34, no. 3, 406–408, 10.1111/j.1365-2230.2008.02898.x, 2-s2.0-62449242746. 19120396

[23] Ryu H. R. , Lee D. W. , Choi H. J. , Kim J. H. , and Ahn H. , Scalp Metastasis of Advanced Gastric Cancer, Archives of Craniofacial Surgery. (2021) 22, no. 3, 157–160, 10.7181/acfs.2021.00129. 34225408 PMC8257450

[24] Menghani S. V. , Barbosa A. , Sagerman P. , Beal M. W. , and Scott A. , Gastric Cardia Adenocarcinoma With Metastasis to the Scalp: A Case Report, Cureus. (2020) 12, no. 1, e6781, 10.7759/cureus.6781. 32140341 PMC7045980

[25] Namikawa T. , Marui A. , Yokota K. , Kawanishi Y. , and Munekage M. , Frequency and Therapeutic Strategy for Patients With Ovarian Metastasis From Gastric Cancer, Langenbeck′s Archives of Surgery. (2022) 407, no. 6, 2301–2308, 10.1007/s00423-022-02543-3. 35551466

[26] Buerba-Vieregge H. H. , Fernández-Ferreira R. , Soberanis-Piña P. D. , De la Peña-López I. R. , and Navarro-García L. M. , Breast Metastasis of Gastric Signet Ring Cell Carcinoma: A Case Report and Literature Review, Case Reports in Oncology. (2021) 14, no. 1, 165–172, 10.1159/000510938. 33776699 PMC7983628

[27] Li R. , Li J. , Wang X. , Liang P. , and Gao J. , Detection of Gastric Cancer and Its Histological Type Based on Iodine Concentration in Spectral CT, Cancer Imaging. (2018) 18, no. 1, 10.1186/s40644-018-0176-2, 2-s2.0-85056374073. PMC6230291 30413174

[28] Zhang L. W. , Wang W. J. , Chen T. , and Xu R. H. , Cutaneous Metastasis From Gastric Carcinoma, Cleveland Clinic Journal of Medicine. (2023) 90, no. 9, 533–534, 10.3949/ccjm.90a.22085. 37657833

[29] Yao G. L. , Tao Y. J. , and Fan Y. G. , Cutaneous Metastasis From Gastric Cancer: Manifestation, Diagnosis, Treatment and Prognosis, European Journal of Surgical Oncology. (2024) 50, no. 2, 107939, 10.1016/j.ejso.2023.107939, 38219697. 38219697

[30] Ben Ismail I. , Sghaier M. , Jouini R. , Rebii S. , and Zoghlami A. , Skin Metastasis Originating From a Gastric Adenocarcinoma, ANZ Journal of Surgery. (2023) 93, no. 5, 10.1111/ans.18359. 37226663

[31] Pliakou E. , Lampropoulou D. I. , Nasi D. , and Aravantinos G. , Skin Metastases From Gastric Cancer, a Rare Entity Masquerading as Erysipelas: A Case Report, Molecular and Clinical Oncology. (2022) 16, no. 6, 10.3892/mco.2022.2543. PMC9112402 35620210

[32] Subramaniam T. , Sister Mary Joseph Nodule and the Mystery Behind Its Nomenclature, Clinical Anatomy. (2022) 35, no. 2, 200–203, 10.1002/ca.23815. 34851538

[33] Kwee R. M. and Kwee T. C. , Imaging in Local Staging of Gastric Cancer: A Systematic Review, Journal of Clinical Oncology. (2007) 25, no. 15, 2107–2116, 10.1200/JCO.2006.09.5224, 2-s2.0-34249950035. 17513817

[34] Shan L. , Ying J. , and Lu N. , HER2 Expression and Relevant Clinicopathological Features in Gastric and Gastroesophageal Junction Adenocarcinoma in a Chinese Population, Diagnostic Pathology. (2013) 9, no. 8, 10.1186/1746-1596-8-76, 2-s2.0-84877117691. PMC3655831 23656792

[35] Bang Y. J. , Van Cutsem E. , Feyereislova A. , Chung H. C. , and Shen L. , ToGA Trial Investigators. Trastuzumab in Combination With Chemotherapy Versus Chemotherapy Alone for Treatment of HER2-Positive Advanced Gastric or Gastro-Oesophageal Junction Cancer (ToGA): A Phase 3, Open-Label, Randomised Controlled Trial, Lancet. (2010) 376, no. 9742, 687–697, 10.1016/S0140-6736(10)61121-X, 2-s2.0-77956262693, 20728210. 20728210

[36] Sohn K. M. , Lee J. M. , Lee S. Y. , Ahn B. Y. , and Park S. M. , Comparing MR Imaging and CT in the Staging of Gastric Carcinoma, American Journal of Roentgenology. (2000) 174, no. 6, 1551–1557, 10.2214/ajr.174.6.1741551, 2-s2.0-0034076877. 10845479

[37] Smyth E. , Schöder H. , Strong V. E. , Capanu M. , and Kelsen D. , A Prospective Evaluation of the Utility of 2-Deoxy-2-[(18) F]Fluoro-D-Glucose Positron Emission Tomography and Computed Tomography in Staging Locally Advanced Gastric Cancer, Cancer. (2012) 118, no. 22, 5481–5488, 10.1002/cncr.27550, 2-s2.0-84868192110. 22549558

[38] Riihimäki M. , Hemminki A. , Sundquist K. , Sundquist J. , and Hemminki K. , Metastatic Spread in Patients With Gastric Cancer, Oncotarget. (2016) 7, no. 32, 52307–52316, 10.18632/oncotarget.10740, 2-s2.0-84982299764. 27447571 PMC5239553

[39] Lordick F. , Carneiro F. , Cascinu S. , Fleitas T. , and Haustermans K. , Gastric Cancer: ESMO Clinical Practice Guideline for Diagnosis, Annals of Oncology. (2022) 33, 1005–1020, 10.1016/j.annonc.2022.07.004. 35914639

[40] Ter Veer E. , Creemers A. , de Waal L. , van Oijen M. G. H. , and van Laarhoven H. W. M. , Comparing Cytotoxic Backbones for First-Line Trastuzumab-Containing Regimens in Human Epidermal Growth Factor Receptor 2-Positive Advanced Oesophagogastric Cancer: A Meta-Analysis, International Journal of Cancer. (2018) 143, no. 2, 438–448, 10.1002/ijc.31325, 2-s2.0-85043719724. 29451302

[41] Kirchberger M. C. , Unusual Presentation of a Cutaneous Metastasis in the Face Arising From Gastric Cancer: A Case Report, SAGE Open Medical Case Reports. (2018) 6, 2050313X18795080, 10.1177/2050313X18795080, 30214808. PMC6134480 30214808

[42] Şahin M. , Ekinci F. , Çelik C. , Temiz P. , and Erdoğan A. P. , A Rare Case Report of Skin Metastasis in Gastric Cancer, Journal of Gastrointestinal Cancer. (2021) 52, no. 3, 1156–1158, 10.1007/s12029-021-00603-3, 33635503. 33635503

[43] Demircioğlu D. , Öztürk Durmaz E. , Demirkesen C. , and Şahin S. , Livedoid Cutaneous Metastasis of Signet-Ring Cell Gastric Carcinoma, Journal of Cutaneous Pathology. (2021) 48, no. 6, 785–788, 10.1111/cup.13969. 33476049

[44] Namikawa T. , Munekage E. , Munekage M. , Maeda H. , and Yatabe T. , Subcutaneous Metastasis Arising From Gastric Cancer: A Case Report, Molecular and Clinical Oncology. (2017) 6, no. 4, 515–516, 10.3892/mco.2017.1175. 28413658 PMC5374947

