# Supplementary material for: Scalp and Neck Metastasis From HER2‐Positive Gastric Cancer: A Case Report and Literature Review
Source: Case Rep Oncol Med. 2026 May 23;2026:9995795. doi: 10.1155/crom/9995795 (PMC13197652; doi:10.1155/crom/9995795)
Supplement: Supplementary file 1 — Supporting Information Additional supporting information can be found online in the Supporting Information section. Supporting Information. [file CROM-2026-9995795-s001.pdf]

CARE Checklist of information to include when writing a case report

CARE case report guidelines

(cc) BY-NC-ND

Topic	Item	Checklist item description	Reported on Line
**Title**	**1**	The diagnosis or intervention of primary focus followed by the words "case report"	Line 1,2
**Key Words**	**2**	2 to 5 key words that identify diagnoses or interventions in this case report, including "case report"	Line 48
**Abstract (no references)**	**3a**	Introduction: What is unique about this case and what does it add to the scientific literature?	Line 34-38
	**3b**	Main symptoms and/or important clinical findings	Line 39-41
	**3c**	The main diagnoses, therapeutic interventions, and outcomes	Line 42-44
	**3d**	Conclusion—What is the main "take-away" lesson(s) from this case?	Line 45-47
**Introduction**	**4**	One or two paragraphs summarizing why this case is unique (**may include references**)	Line 50-62 (2-7)
**Patient Information**	**5a**	De-identified patient specific information.	Line 68-69
	**5b**	Primary concerns and symptoms of the patient.	Line 70-73
	**5c**	Medical, family, and psycho-social history including relevant genetic information	Line 68
	**5d**	Relevant past interventions with outcomes	Line 69
**Clinical Findings**	**6**	Describe significant physical examination (PE) and important clinical findings.	Line 72-73
**Timeline**	**7**	Historical and current information from this episode of care organized as a timeline	Line 74-75
**Diagnostic Assessment**	**8a**	Diagnostic testing (such as PE, laboratory testing, imaging, surveys).	Line 74-77
	**8b**	Diagnostic challenges (such as access to testing, financial, or cultural)	Line 78-79
	**8c**	Diagnosis (including other diagnoses considered)	Line 80
	**8d**	Prognosis (such as staging in oncology) where applicable	Line 81
**Therapeutic Intervention**	**9a**	Types of therapeutic intervention (such as pharmacologic, surgical, preventive, self-care)	Line 81-82
	**9b**	Administration of therapeutic intervention (such as dosage, strength, duration)	Line 83
	**9c**	Changes in therapeutic intervention (with rationale)	Line 84-85
**Follow-up and Outcomes**	**10a**	Clinician and patient-assessed outcomes (if available)	Line 86-87
	**10b**	Important follow-up diagnostic and other test results	Line 87-88
	**10c**	Intervention adherence and tolerability (How was this assessed?)	Line 86
	**10d**	Adverse and unanticipated events	Line 86
**Discussion**	**11a**	A scientific discussion of the strengths AND limitations associated with this case report	Line 90-143
	**11b**	Discussion of the relevant medical literature **with references**.	Line 90-194 (12–24)
	**11c**	The scientific rationale for any conclusions (including assessment of possible causes)	Line 199-200
	**11d**	The primary "take-away" lessons of this case report (without references) in a one paragraph conclusion	Line 199-203
**Patient Perspective**	**12**	The patient should share their perspective in one to two paragraphs on the treatment(s) they received	Line 80
**Informed Consent**	**13**	Did the patient give informed consent? Please provide if requested	**Yes** [X] **No** [ ]
